# Conformational Dynamics of Bacteriochlorophyll *c* in Chlorosomes from the *bchQ* Mutant of *Chlorobaculum tepidum*

**DOI:** 10.1021/acs.jpcb.4c04731

**Published:** 2025-02-17

**Authors:** Lolita Dsouza, Karthick Babu Sai Sankar Gupta, Xinmeng Li, Vesna Erić, Yusen Luo, Annemarie Huijser, Thomas L. C. Jansen, Francesco Buda, Alfred R. Holzwarth, Donald A. Bryant, Andrei Gurinov, G. J. Agur Sevink, Huub J. M. de Groot

**Affiliations:** † Leiden Institute of Chemistry, Leiden University, Einsteinweg 55, 2300 RA Leiden, The Netherlands; ‡ Department of Chemistry and Hylleraas Centre for Quantum Molecular Sciences, University of Oslo, 0315 Oslo, Norway; § Zernike Institute of Advanced Materials, University of Groningen, Nijenborgh 3, 9747 AG Groningen, The Netherlands; ∥ MESA+ Institute for Nanotechnology, University of Twente, 7500 AE Enschede, The Netherlands; ⊥ Max Planck Institute for Chemical Energy Conversion, Stiftstraße 34-36, 45470 Mülheim an der Ruhr, Germany; # Department of Biochemistry and Molecular Biology, The Pennsylvania State University, University Park, Pennsylvania 16802, United States; ∇ NMR Spectroscopy, Bijvoet Center for Biomolecular Research, Utrecht University, Padualaan 8, 3584 CH Utrecht, The Netherlands

## Abstract

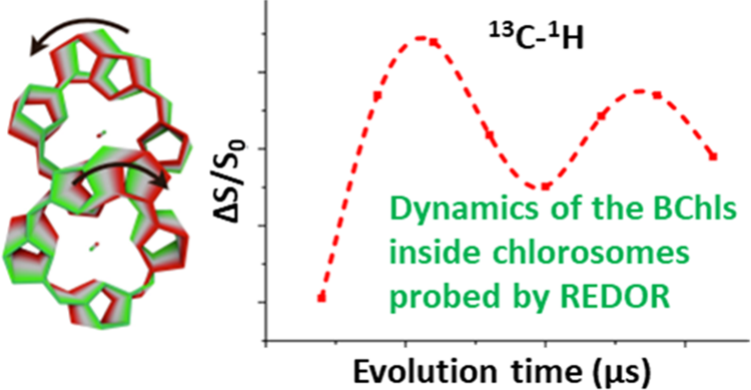

In contrast to the common viewpoint that bacteriochlorophyll (BChl) motion is largely absent within the chlorosome assembly, physics-based modeling points to a crucial role of the nanoscale librational motion of the macrocycle for the transfer of excitons. To elucidate this motion experimentally, compositional uniformity and high sensitivity are required. We focused on uniformly ^13^C labeled chlorosome preparations from the *bchQ* mutant *Chlorobaculum tepidum* with significantly enhanced structural homogeneity. The librational motion is characterized using Rotational Echo DOuble Resonance (REDOR), and in addition, the impact of temperature on specific functionalities within BChl molecules is studied with 1-dimensional and 2-dimensional dipolar and scalar-based MAS NMR measurements. Results show the gradual freezing of the tails and side chains of the BChls with decreasing temperature. However, the librational motion analyzed by measuring the 5C–H dipolar coupling strength obtained from REDOR data sets persists at different temperatures. REDOR simulations show a close match to the experimental dephasing frequency of oscillation for a dipolar coupling strength of 17.5 ± 0.5 kHz which is considerably less than the dipolar coupling strength of 22.7 kHz in the rigid limit. Following a two-site jump model, we arrive at an estimate for BChl libration sampling at an angle of θ = 48 ± 4°, corroborating that the macrocycle indeed experiences significant librational motion on a time scale that is short compared to the NMR measurement time. This finding is in full quantitative support of the dominant rotational motion exhibited by the BChl macrocycle estimated from early MD simulations.

## 1 Introduction

The first step of photosynthesis involves capturing photons and converting them to usable chemical energy. In green bacteria, light harvesting is carried out by chlorosomes.^1^ The captured light energy is transferred to the FMO complex through the baseplate and then to the reaction center, where charge separation occurs.^2^ The green sulfur bacterium *Chlorobaculum tepidum* is particularly effective at this conversion in low light conditions, present hundreds of meters below the surface of the water in the Black Sea.^3−5^

Chlorosomes are antenna organelles that are on average 100–200 nm in length and 50–60 nm in diameter, containing bacteriochlorophyll *c* (BChl *c*) as the main pigment. There is converging evidence that the BChl *c* in chlorosomes self-assembles into *syn-anti* parallel stacks that form curved sheets and concentric tubes, without the involvement of proteins in the aggregation.^1,6,7^ The suprastructure is induced and stabilized by interactions between the chromophores, such as coordination of Mg^2+^ with the 3^1^-hydroxyl groups between the two neighboring BChls, hydrogen bonding between 13^1^ carbonyl groups and 3^1^ hydroxyl groups, π–π stacking interactions between chromophores, as well as hydrophobic interactions of the tails.^8,9^ The structure and composition of chlorosomes vary depending on the growth conditions, making them challenging to understand. Because chlorosomes are heterogeneous and radiation sensitive, they are not amenable to study by X-ray or other diffraction methods. Various techniques, such as cryo-EM, solid-state NMR, and optical spectroscopy, have been combined to provide an experimental basis for structure determination, leading to a bottom-up, multiscale computational model of the chlorosome as a tubular plastic crystal composed of *syn-anti* parallel stacks.^9−12^

The structure of the BChls in chlorosomes from a *bchQ* null mutant of *C. tepidum* has recently been determined by Dsouza *et al*. For convenience, we shall refer to these as "*bchQ* chlorosomes". It is the starting point of the current study because it is primarily composed of a single [8-Ethyl, 12-Ethyl] BChl *c* homolog, rendering it significantly more homogeneous than the wild type (WT) chlorosomes from this organism.^13^ In the earlier work, we obtained preliminary insights on the dynamics of BChls at room temperature.^12,13^ We observed rigid stacks, but the tails attached to the BChls and tetrapyrrole side chains showed some flexibility. The rigidity of the stacks would align with the plastic crystalline character due to the limited librational motion proposed by modeling.^12,13^ The specific aims of this study are to investigate if the librational motion of the BChl *c* molecules is observed for *bchQ* chlorosomes within the rigid *syn-anti* parallel stacking motifs, determine with Magic Angle Spinning (MAS) NMR the angle over which limited librational dynamics of the oriented BChl *c* macrocycles occurs, and show that libration persists over the accessible temperature range. We also show that the libration contrasts with the dynamic behavior of the farnesyl tail and side chains of the tetrapyrrole ring, which can be frozen by decreasing the temperature.

In our previous study, we have shown that it is possible to resolve site-specific dynamics of chlorosomes using MAS NMR ^1^H–^13^C polarization transfer dynamic spectral editing (DYSE).^13^ This method helped us to reduce spectral crowding by selectively detecting molecules with dynamics within a specific frequency range while filtering out other signals.^14,15^ In our current study, we used similar experimental techniques but at varying temperatures. To further resolve the signals, we made use of two-dimensional (2-D) measurements to determine if there is any effect on the rotational dynamics of the macrocycles within the stack. In addition, we used Rotational Echo Double Resonance (REDOR) to quantify the dipolar coupling strength. This measurement provides direct access to the amplitudes of librational motion via the scaling of dipolar couplings through partial motional averaging.^16^

The *syn-anti* BChl *c* parallel stacks in chlorosomes are held together by nonbonding pi-pi stacking interactions and distinct hydrogen bonding configurations that are distributed randomly over the structure.^17^ The plastic crystalline character is thought to be due to a rotational degree of freedom of individual BChl *c* pigments within a quasi-crystalline packing. This combination of static and dynamic characteristics is believed to generate dynamic level crossings in the exciton manifold of states and transient quantum coherence for ultrafast interconversion of states, which is the cause of the ultrafast energy transfer in chlorosomes.^12,18^ To investigate the rotational motion of the BChl *c* within the stack, we utilize a direct measurement of the dipolar coupling between a C–H pair at a *meso*-position of the macrocycle using REDOR. The 5C–H *meso*-position is chosen because it is well resolved from other carbons, which is a favorable condition for REDOR measurements. Librational movement of the macro aromatic cycle in the BChl *c* is expected to partially average the 5C–H heteronuclear dipolar coupling.^19^ Since REDOR gives direct access to dipolar coupling strength, we thus probe the dipolar coupling and compare it to the value expected in the case of complete rigidity.^16,20,21^ REDOR is a robust technique because of its ease of experimental setup, limited susceptibility to RF field inhomogeneities, and easy extraction of the dipolar coupling strength from the NMR data. It is often used as a technique for the measurement of dipolar coupling strength and dynamics.^22−30^

The application of REDOR is not straightforward for the study of abundant, strongly coupled ^1^H spins.^16,21^ However, recently, REDOR was successfully applied in combination with rapid spinning to measure many individual ^1^H–^13^C dipolar couplings and address their scaling in uniformly ^13^C labeled model protein with REDOR simulations of single C–H spin pairs.^31^ For our system, a sparse density of protons for the highly unsaturated BChl macrocycles in the vicinity of the 5C–H moiety offers a window of opportunity to use REDOR for probing C–H dipolar coupling strength without deuteration.^32^ Due to the relatively sparse proton network, the impact of homonuclear dipolar contributions on determining accurate dipolar coupling strength is limited.^31^

## 2 Materials and Methods

### 2.1 Sample Preparation

*C. tepidum* bacteria were grown in high-light conditions according to procedures given by Tian *et al*.^33^ The chlorosome sample for NMR was prepared by the methods described by Dsouza *et al*. and was filled in 1.3 and 3.2 mm^2^ zirconium rotors for NMR measurements.^13^

### 2.2 Solid-State NMR Measurements

MAS NMR experiments were conducted on freshly prepared *bchQ* chlorosomes uniformly labeled with the ^13^C isotopes. The magnet AV-750 MHz (17.1 T) was equipped with a state-of-the-art Avance Neo console (Bruker, Billerica, MA). The 1-D and 2-D measurements were carried out using a 3.2 mm E-Free probe equipped with HCN channels. The spinning frequencies used for the measurements are 20 and 11 kHz, respectively, at the magic angle. The temperature range for investigating the dynamics was measured with a thermometer in the probe and was 207–277 K, which corresponds to sample temperatures of *ca*. 235 to 282 K. 1-D Cross-Polarization (CP) and Insensitive Nuclei Enhanced by Polarization Transfer (INEPT) measurements were performed with 1k scans. The recycle delay was set to 2 s for CP, while the delay periods were set to 1.75 and 1.15 ms for INEPT. The proton π/2 pulse length for CP was 2.5 μs, corresponding to a 100 kHz rf amplitude. ^13^C–^13^C Proton-Driven Spin Diffusion (PDSD) NMR data were collected with 256 scans for each trace in the *t*_1_ dimension, using a ^13^C–^13^C mixing time of 25 ms at temperatures of 235 and 282 K. SPINAL-64 decoupling was applied during the *t*_2_ acquisition period.^34^ A ^13^C–^13^C INEPT-TOtal through Bond correlation SpectroscopY, also known as (TOBSY) experiment shown in Figure 3b was performed with the sample in a 3.2 mm rotor at the magic angle in a Bruker Avance III 700 (16.4 T) spectrometer, employing a TOBSY mixing time of 6.5 ms and *P*9_6_^1^-TOBSY mixing symmetry was used.^35^

The temperature was calibrated from the thermosensitive chemical shift of a sample of KBr. Signals from ^79^Br were produced and gathered at a frequency of 188 MHz through π/2 direct excitation and acquisition with a single scan. The temperature within the MAS stator and rotor was controlled by adjusting the external VT gas flow. Temperature calculations were performed according to the methods outlined in the ref (36).

^13^C{^1^H}REDOR data were recorded from a 1.3 mm rotor spinning at 50 kHz at a probe display temperature of 255, 268, and 282 K which corresponds to sample temperatures of *ca*. 288, 299, and 313 K, respectively, at the magic angle in a Bruker AV-750 (17.4 T).^36^ The CPMAS spectra were obtained with a π/2 pulse width of 1.5 μs, a contact time of 2 ms, and a recycle delay of 2 s. The pulse widths for the ^13^C and ^1^H π pulses during REDOR were 10 and 3.0 μs, respectively. The pulse sequence used for REDOR can be found in the Supporting Information in Figure S1. For phasing, the XY-8 scheme was used.^37^ The data were processed and analyzed using TOPSPIN and the simulations were conducted using SIMPSON.^38^ To estimate the Euler angles for the BChl, SIMMOL VMD was used.^24,39^ The estimated Euler angles were 0, 98.58, and −25.11. For the SIMPSON simulations, the rep 320 crystal file was used for powder averaging.^38^

## 3 Results

### 3.1 Temperature Dependence of the BChls in *bchQ* Chlorosomes Using 1-D NMR Spectroscopy

**Figure 1:**
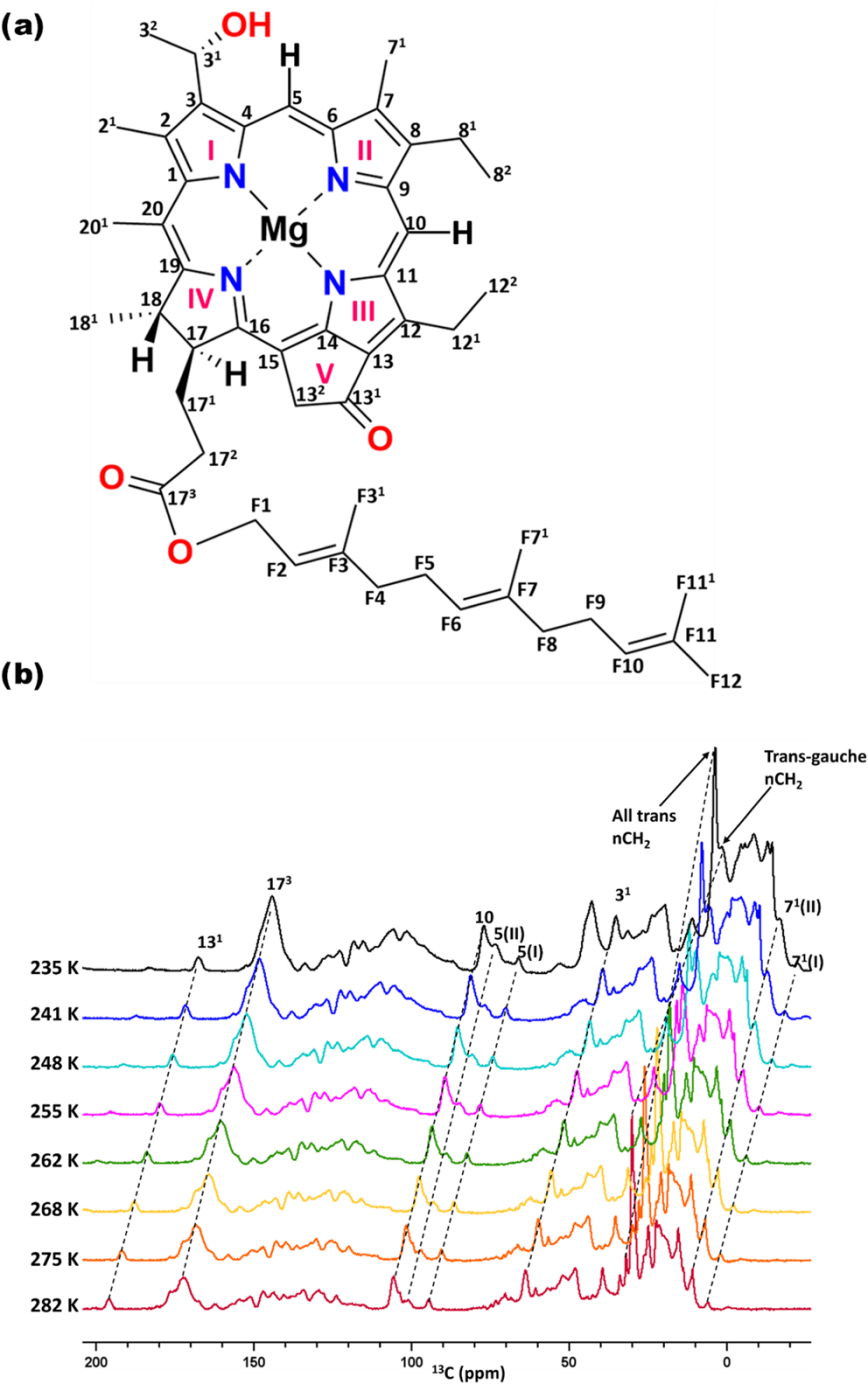
(a) Chemical structure of [8Et, 12 Et] BChl *c* with IUPAC numbering. Figure 1. (b) stacked plot of CPMAS spectra collected at 282 K (Red), 275 K (Orange), 268 K (Yellow), 262 K (Green), 255 K (Pink), 248 K (Cyan), 241 K (Blue), and 235 K (Black) while spinning at 20 kHz. A horizontal and vertical offset of 10 ppm was applied to every trace to improve visibility.

MAS NMR is a powerful and unique technique for studying the structure and dynamics of supramolecular aggregates.^40,41^ In this paper, data sets were collected at temperatures ranging from 235 to 282 K using CP and INEPT techniques. Figure 1b shows CPMAS spectra collected at different temperatures. The aliphatic region between 0 and 60 ppm mainly contains overlapping farnesyl tail carbon resonances and the response from the side chains of the BChl macrocycle. The 1-D spectra display peak splitting for the 7^1^C and 5C, which serve as reference points to illustrate the two signal conformations within the system that have been attributed to a major component (I) of H-bonded and a minor component (II) of non-H-bonded BChls.^17^ Signals in the 70–80 ppm region are attributed to galactolipids like monogalactosyldiacylglycerol (MGDG).^7,42^ The aromatic region includes methine carbon resonances for the 5 and 10th carbon atoms, while the 170–200 ppm region contains carbonyl signals. A small shoulder at 176 ppm, which is associated with the 17^3^C response at 173 ppm, could be attributed to a carbonyl signal from proteins in the lipid envelope. The BChl 13^1^ carbonyl peak is at 196 ppm. With decreasing temperature, CP intensities gradually increase due to enhanced CP efficiency caused by decreased dynamics of the molecules, as shown in Figure 1b. The ^13^C in the lipids surrounding the BChls in the chlorosome show a broad response around 30 ppm, which includes unresolved resonances from CH_2_ groups and a weak signal at 32 ppm. Lipids resulting in different ^13^C chemical shifts for the acyl chain carbons can adopt *all-trans* or *trans–gauche* conformations. The small weak signal at 32 ppm corresponds to CH_2_ carbons of lipids in the *all-trans* conformation, while the main peak at 30 ppm represents the carbons of lipids in the *trans–gauche* conformation. This type of behavior was also demonstrated by Azadi *et al*. for thylakoid membranes.^43^ Unlike other CP signal intensities that decrease at higher temperatures due to increased molecular dynamics, the intensity of the *trans–gauche* lipid peak at 30 ppm increases with the temperature. This observed increase may suggest the occurrence of *all-trans* to *trans–gauche* isomerization at elevated temperatures for chlorosome lipids.^44^

**Figure 2:**
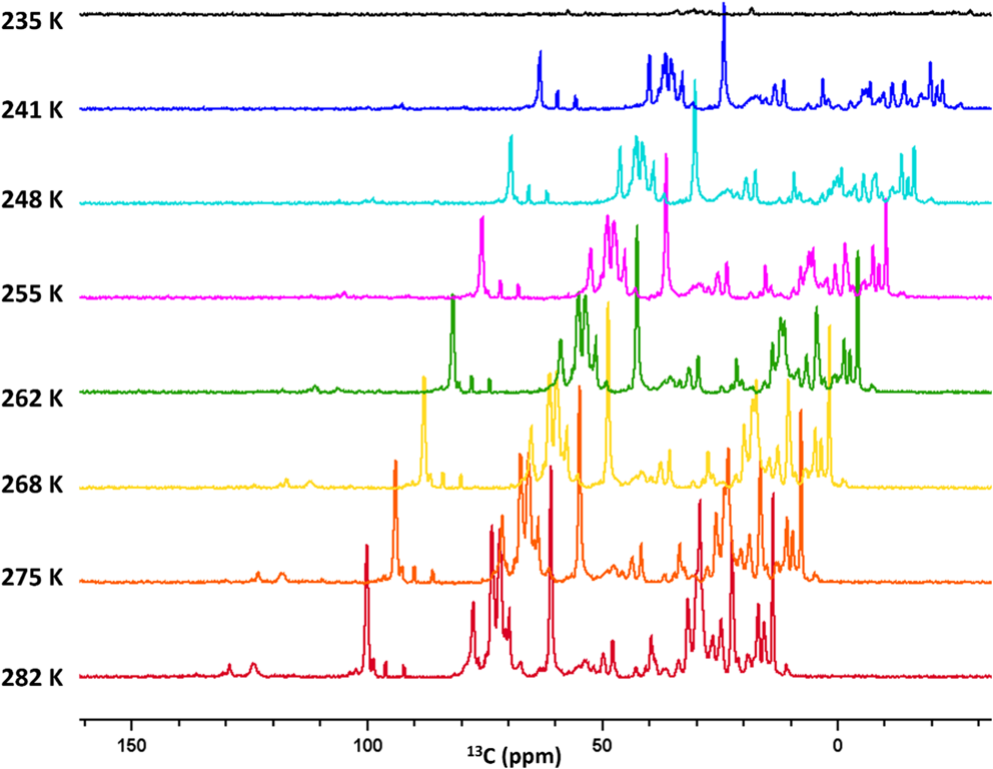
Overlaid INEPT spectra at 282 K (Red), 275 K (Orange), 268 K (Yellow), 262 K (Green), 255 K (Pink), 248 K (Cyan), 241 K (Blue), and 235 K (Black) recorded in a 3.2 mm rotor spinning at 20 kHz. A 10 ppm horizontal and vertical offset has been applied to the traces to improve visibility.

Figure 2 shows INEPT data sets collected at the same temperatures as the CP data sets shown in Figure 1b. The signals that are less enhanced by CP are enhanced by INEPT at higher temperatures. We observe the freezing of these signals at 235 K. At this temperature, the differences between INEPT intensities and CPMAS signal strengths are indicative of restricted mobility arising from the immobilization of the BChl mobile segments and the lipids surrounding it. As the temperature increases, the INEPT intensities gradually increase, signifying an expansion in the fraction of BChl mobile segments and mobile lipids surrounding them. This observation underscores the impact of the temperature on the dynamic behavior of carbon resonances and the associated mobility of molecular constituents within the system. For the INEPT signals that are present at room temperature, the intensity is reduced as the temperature is decreased. They can be attributed to the resonances from the aliphatic region, including carbon resonances from the farnesyl tails, such as F1, F2, F3^1^, F4, F5, F7, F7^1^, F8, F9, F10, F11, F11^1^, F12, side chains attached to the BChl macrocycle, such as 2^1^, 3^2^, 7^1^, 20^1^, and the lipids from the envelope surrounding the BChl supramolecular assemblies.^13^

### 3.2 Two-Dimensional NMR Spectroscopy on Chlorosomes

2-D homonuclear dipolar measurements are commonly used in the field of solid-state NMR, since they help in assigning signals and obtaining geometrical information about a molecule by recording the transfer of polarization among spins.^45^ To validate the 1-D findings, we performed 2-D NMR to observe the effect of temperature as it provides better resolution compared to its 1-D counterparts. The dynamics of BChls inside chlorosomes were investigated using a homonuclear ^13^C–^13^C spin diffusion-based experiment, where polarization is transferred by utilizing dipolar couplings.^46^ PDSD is a versatile technique when the sample is uniformly labeled and dipolar truncation is present.^45^

**Figure 3:**
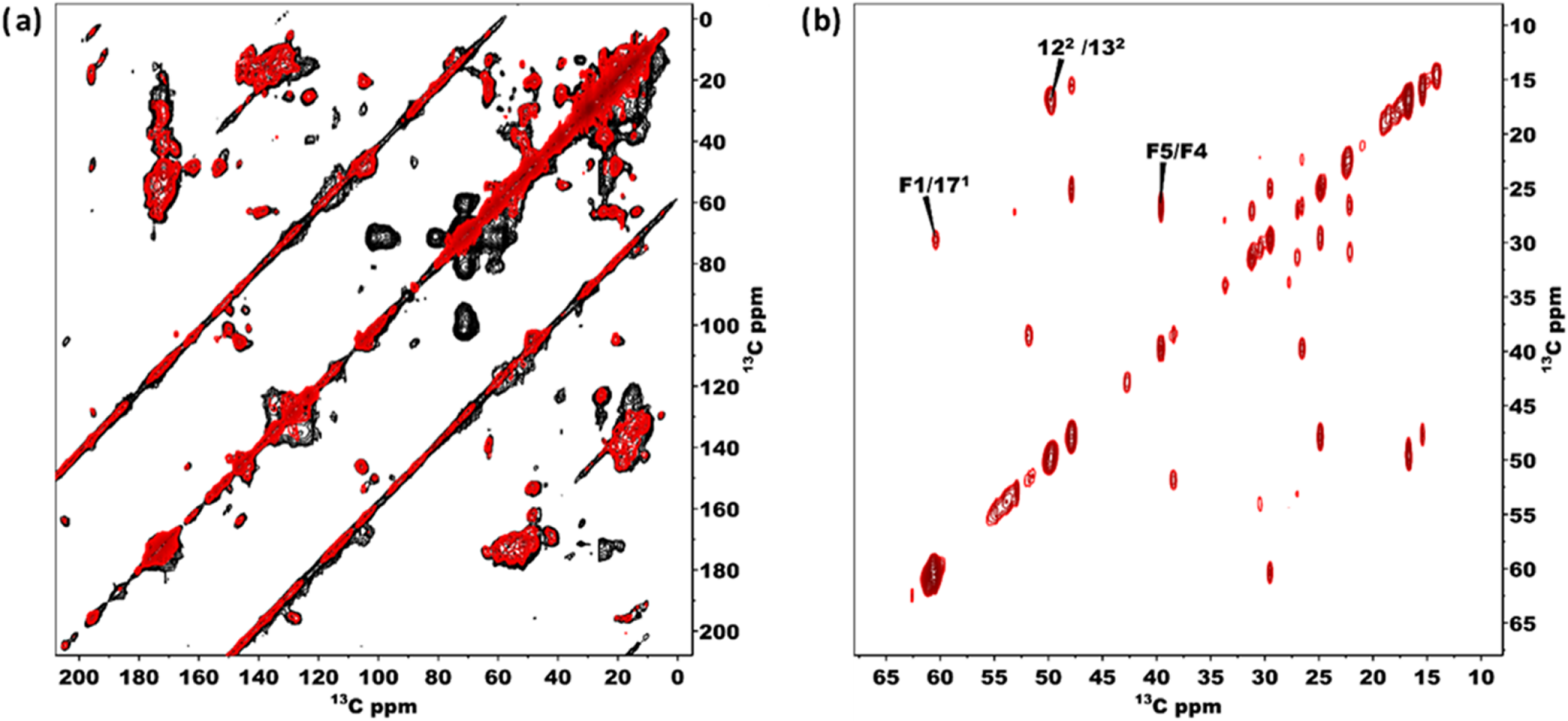
(a) Overlaid ^13^C–^13^C PDSD spectra of chlorosomes obtained at 25 ms mixing time at 235 K (Black) and 282 K (Red). It shows cross peaks for immobilized segments. Figure 3. (b) is a ^13^C–^13^C INEPT-TOBSY spectrum of chlorosomes from *C. tepidum* was obtained at 6.5 ms mixing time at 282 K, showing cross peaks for dynamic residues.

Figure 3a shows the PDSD data sets collected from a uniformly labeled *bchQ* chlorosome sample preparation at 282 (Red) and 235 K (Black) with an 11 kHz spinning frequency. With PDSD, the radio frequency field is switched off during the mixing time, and transfer proceeds faster at a lower spinning frequency.^45^ In PDSD, the transfer of polarization between carbon nuclear spins proceeds by an overlap between ^13^C signals, which is facilitated by the dipolar broadening from the surrounding protons. The rate of this transfer is dependent on the dipolar interactions between the ^13^C spins and their interaction with the surrounding protons. Interestingly, at lower temperatures many cross peaks show increased intensity. In addition, cross peaks are observed that were not present at higher temperatures, for ^13^C resonances from the tails and side chains of the BChl macrocycle with similar chemical shifts, such as F7/F12, F11/F12, F6/F3^1^, F1/F2, 3^2^/2^1^, 17^3^/F4, 17^1(2)^/17(18) F9/F11, F10/F7^1^, and 20/17^1^. The increase in cross-peak intensities at lower temperatures is attributed to the freezing of the dynamics that modulate the strength of the dipolar couplings. In contrast, increasing temperatures enhance the mobility in the system. This leads to partial motional averaging of the dipolar couplings, resulting in weaker dipolar couplings and fewer cross peaks for tail carbon resonances and side chains attached to the BChl macrocycle.^47^

The two H-bonded and non-H-bonded BChl 5C–H resonances at 95 and 101 ppm, respectively, appear preserved on the millisecond time scale of the PDSD experiments in Figure 3a. Chemical exchange between H-bonded and non-H-bonded BChls and associated line-broadening, cross-peaks, or averaging over the two signals was not detected over the temperature studied. This leads us to conclude that the H-bonded and non-H-bonded fractions are conserved on all NMR time scales.

We used a *J*-based INEPT-TOBSY technique to selectively detect molecules and fragments subject to rapid motion.^48^Figure 3b shows an INEPT-TOBSY data set recorded at 282 K, which is the temperature where the INEPT signal was strongest in the 1-D spectra (see Figure 2). In this spectrum, we observe intramolecular cross-peaks between F1/17^1^, 12^2^/13^2^, and F5/F4. The unassigned cross peaks may be attributed to lipids surrounding the BChl aggregates. These cross peaks are not visible in the 2-D spectra based on dipolar interactions at 282 K. However, they do appear at lower temperatures (Figure 3a). A comparison of both spectra reveals that most of the cross-peaks are visible in dipolar-based spectra, while only a few are seen in *J*-based spectra. This suggests that the rings and stacks are crystalline, and side chains and tails show some flexibility, which supports our idea of the plastic crystal nature of the concentric tubular BChl self-assemblies in chlorosomes that are separated by tail regions with high flexibility between the tubes.

### 3.3 Probing Rotational Motion of the Macrocycle

A plastic crystal is a material that has a local orientational or conformational degree of freedom.^12^ Extensive modeling of chlorosome assemblies points to libration, rotational motion of the BChl macrocycles, as a distinctive dynamic mode of central importance for the light-harvesting function.^12,17,18^ This libration is a persistent and partially restricted motion that cannot be probed directly using the PDSD or TOBSY sequence due to limitations in the time resolution of NMR. Therefore, to resolve this motion, we investigated the strength of the 5C–H dipolar coupling with ^13^C{^1^H} REDOR dephasing experiments.^32,49−52^ This technique allows one to estimate how libration reduces the dipolar coupling by partial averaging. We see from the CP versus INEPT results that the dynamics in other parts of the BChls in the chlorosome are frozen and show strong temperature dependence in the considered temperature range but that libration persists for all temperatures. Consequently, we performed REDOR at three different temperatures.

**Figure 4:**
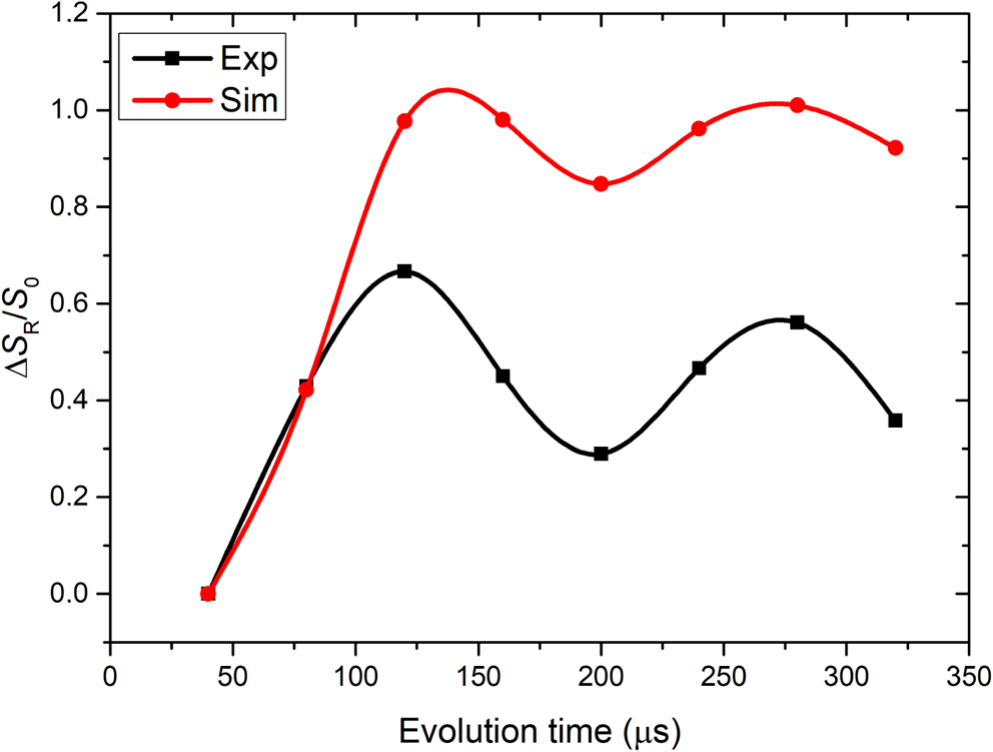
Shows the ^13^C{^1^H} REDOR dephasing as a function of time in μs for the 5C–H pair (see Figure 1a for the chemical structure of BChl) at 288 K where the carbon resonates at 95 ± 0.5 ppm shown with a cubic spline (black curve) through the experimentally obtained data (black filled squares). The simulations are shown in red (filled red circles and cubic spline interpolation curve) where the frequency of oscillation matches the experimental curve.

REDOR operates by reintroducing the heteronuclear dipolar couplings that are averaged out due to MAS through the application of π pulses every half a rotor period to refocus the ^1^H–^13^C heteronuclear dipolar coupling. This results in dipolar dephasing of the ^13^C, which causes a decrease in the intensity of the observed signal.^53^ The data are collected through two consecutive experiments, one with and one without π pulses, resulting in *S*_R_ and *S*_0_ data sets that represent dipolar dephasing and natural dephasing, respectively. The variations in intensity for the difference between these two data sets allow one to extract the dipolar coupling strength by comparing it with simulations of the REDOR process. Figure 4 displays the experimental and simulated ^13^C{^1^H} REDOR dephasing (Δ*S*_R_/*S*_0_) as a function of evolution time in μs for the 5C–H pair while spinning at 50 kHz. The REDOR curves at 299 and 313 K can be found in Figure S4, S5. For the analysis, we focus on the frequency of the dipolar oscillation and compare the experimental and simulated data considering the dephasing curve over a time of up to 350 μs for measuring the directly bonded ^13^C–^1^H spin pair.^32^ We used short evolution times to probe the dipolar coupling for the CH pair with no nearby protons present as to keep the deviations from the ideal oscillation profile minimal.^31^ Longer evolution times are not considered, as the ^13^C–^1^H spin interactions from distant protons become dominant.

The fast internal motions can be described by an order parameter *S = δ*_exp_/*δ*_rigid_, which quantifies the extent of spatial restriction of the motion.^21^ A match between simulated and experimental REDOR dephasing frequency of oscillation is found for a dipolar coupling strength δ_exp_ = 17.5 ± 0.5 kHz. This is considerably less than the dipolar coupling strength δ_rigid_ = 22.7 kHz in the rigid limit, and we obtain *S* = 0.77 ± 0.03. This can be used to estimate a libration angle θ = 48 ± 4° using a two-site jump model following Schanda and Ernst^21^ according to . As a reference rigid limit value for the one bond C–H coupling, we used the previously determined 22.7 kHz corresponding to a C–H bond length of 1.10 Å.^31,54^ The results obtained for 299 and 313 K also match the value for libration angle θ extracted for 288 K, which provides evidence that the libration persists over this temperature range, in line with earlier findings obtained *via* MD simulations.^12,17^

## 4 Discussion

The proposed mechanism for the suprastructure of the BChl pigments to resolve structural frustration is by enabling rotational disorder in the plane of the macrocycle. The two conformations in the system, which are observed in NMR as peak splitting,^7,9^ have been attributed to H-bonded and non-H-bonded BChls and are static on the scale of NMR, *i*.*e*. we see two separate and narrow signals on the diagonal, without cross-peaks from chemical exchange.^12,17,18^

Recent advancements in MAS NMR have shown the significance of utilizing both dipolar and *J*-based pulse sequences to investigate the dynamics of the molecules on different time scales.^55^ We adopted this strategy and successfully gained a comprehensive understanding of the dynamics of chlorosomes, which inspired us to further explore the dynamic rotational disorder of the BChl macrocycles, in particular, the fluctuation about the H-bonded conformation, to make a connection between the experimental and computational studies.^12^

### 4.1 Dynamics in the Chlorosomes According to 1-D NMR Spectroscopy

Most of the NMR signals of chlorosomes are visible in the CP-based spectrum, while only a specific set of signals can be observed in the INEPT *J*-based spectrum.^13^ The INEPT data sets display signals related to large amplitude motions occurring on a picosecond (ps) to nanosecond (ns) time scale, primarily in the aliphatic region of the chlorosome response where carbon resonances from side chains and the farnesyl tail of the BChl macrocycle are present. No abrupt changes in the intensities of CP and INEPT are observed across the temperature range that would suggest a phase transition from freezing. Instead, the temperature curves indicate a gradual increase in the dynamics and an overall rise in site-specific dynamics of the chlorosome BChls at higher temperatures on both fast picosecond to nanosecond and slow millisecond time scales of the fast and the slow BChl components and progressive isomerization of the lipids surrounding the BChl. The lipid dynamics appear to be decoupled from the macrocycle dynamics. The BChl ^13^C response in the CP spectra is well in line with the tightly packed nature of the BChl rings and the plastic crystallinity of the stacks forming the tubular BChl suprastructure in the chlorosomes, where the BChl macrocycles exhibit restricted dynamics in an overall crystalline packing. The BChls in chlorosomes take the form of concentric cylindrical structures, and variations in energy between super-radiant states due to different curvature and hydrogen bonding patterns result in dispersed exciton states.^11^ Due to the dynamics in the system, the exciton states will undergo level crossing and may be prone to quantum instabilities.^56^ As the temperature decreases, the packing of the BChl macrocycles becomes restricted while the libration persists.

### 4.2 Dynamic Study According to Dipolar-Based and Scalar-Based 2-D NMR Spectroscopy

Solid-state 2-D NMR experiments are a reliable and widely used methodology for studying the structure and dynamics of a system. Proton-driven spin diffusion experiments are useful when the sample is fully labeled and are highly effective in detecting small homonuclear ^13^C–^13^C dipolar couplings over long internuclear distances.^45^

Carbons with increased mobility produce weak cross-peaks because the increased mobility decreases effective dipolar couplings. For a molecule to exhibit intense cross peaks in the 2-D MAS spectra, the molecular environment must be rigid and solid.^7^ Our findings revealed that some ring carbon atoms of the BChl pigments in the chlorosome macrocycle and some from the farnesyl tail are not visible in the PDSD spectra due to the millisecond dynamics seen in the BChls. This motion is suppressed when the temperature is lowered, leading to the appearance of cross peaks in the 2-D spectra.

TOBSY is a widely used technique that is combined with dipolar-based experiments to investigate both the dynamics and rigidity of a sample simultaneously.^14,47,57,58^ This technique is utilized to study the through-bond ^13^C–^13^C connectivity, and the results obtained are consistent with the findings from 1-D INEPT measurements, Figure 3b. The improved resolution in the 2-D spectra allows for the differentiation of ^13^C connectivity and, consequently, dynamics. Additionally, the lipids surrounding the chlorosomes are visible in the INEPT based experiments, indicating their prominent level of dynamics.

### 4.3 Probing Rotational Motion of the Macrocycle

In the context of probing fast μs-ps dynamics, several routes are possible, including the above-described 1-D CP and INEPT, relaxation measurements, or estimating the dipolar coupling strength. The reduction in dipolar coupling strength relative to the rigid limit is in line with partial motional averaging due to fast restricted motion.^28^ For XY-4 phase cycling and its extensions, the finite pulses were found to have a minor impact on the dipolar scaling factor.^37^ For the 5C–H in the BChls in chlorosomes, the dipolar coupling was measured for the major component and appears partially averaged with *S* = 0.77 ± 0.03, and we attribute this to the rotational oscillation of the H-bonded BChls in the plane of the macrocycle, which partially averages out the dipolar coupling from *ca*. 22.7 to 17.5 ± 0.5 kHz. A moderate scaling of the order parameter to 0.77 ± 0.03 gives an angle between the main tensor axes at the two extreme positions of θ = 48 ± 4°. This result agrees rather well with the analysis of the libration motion of BChl *c* by molecular dynamics simulations (see SI, Figure S6b).

A limited RF amplitude with the application of π pulses on the ^1^H channel and moderate strength of the heteronuclear dipolar coupling can lead to nonideal REDOR profiles.^31^ The reduction in height of the experimental dephasing curve can be expected for systems with extended homonuclear dipole–dipole couplings, while according to simulation studies the initial part of the dephasing profile shows only minor deviations from the ideal profile.^31^ The utilization of the approach with ^1^H refocusing pulses was favored over the alternative approach with REDOR on the ^13^C nuclei. Due to rapid spinning, the timing of ^13^C REDOR pulse trains is difficult. The longer duration of ^13^C π pulses relative to ^1^H requires much longer REDOR periods to avoid continuous RF over the entire rotor period and leads to low resolution in the REDOR dephasing curves since few data points can be collected.

## 5 Conclusions

We used solid-state NMR spectroscopy on a fully ^13^C labeled system to investigate the dynamics of various parts of a BChl molecule, as well as the rotational mode within the BChls of *bchQ* chlorosomes. Detailed measurement of the dipolar coupling strength allowed us to analyze the anticipated rotational motion of the macrocycle. The temperature-dependent dynamics of other parts of the BChl molecules were analyzed by a combination of NMR methods at different temperatures. Our observations are fully consistent with the tight packing of the macrocycles in rigid stacks forming the tubes, while the side groups and tail exhibit some degree of mobility. From the measurement of the dipolar coupling strength, we determine that BChls experience librational motion between two extremes at an angle of θ = 48 ± 4° within the tightly packed stacks. This finding confirms the plastic crystallinity of BChls within chlorosomes proposed earlier based on computational studies.

## Supporting Information Available

Additional experimental details; simulations of the dephasing profiles for different dipolar coupling strengths; simulations at the different temperatures, and connection to previous physical modeling studies with the obtained experimental results are outlined (PDF)
