# Supplementary material for: Conformational Dynamics of Bacteriochlorophyll c in Chlorosomes from the bchQ Mutant of Chlorobaculum tepidum
Source: J Phys Chem B. 2025 Feb 17;129(8):2129–37. doi: 10.1021/acs.jpcb.4c04731 (PMC11873974; doi:10.1021/acs.jpcb.4c04731)
Supplement: Supplementary file 1 — jp4c04731_si_001.pdf [file jp4c04731_si_001.pdf]

Conformational Dynamics of Bacteriochlorophyll *c* in Chlorosomes from the *bchQ* Mutant of *Chlorobaculum Tepidum*

Lolita Dsouza[1], *Karthick Babu Sai Sankar Gupta*[1], *Xinmeng Li*[1,2], *Vesna Erić*[3], *Yusen Luo*[4], *Annemarie Huijser*[4], *Thomas L.C. Jansen*[3], *Francesco Buda*[1], *Alfred R. Holzwarth*[5], *Donald A. Bryant*[6], *Andrei Gurinov*[7], *G.J. Agur Sevink*[1*], *Huub J.M. de Groot*[1*]

[1] Leiden Institute of Chemistry, Leiden University, Einsteinweg 55, 2300 RA, Leiden, the Netherlands.

[2] Department of Chemistry and Hylleraas Centre for Quantum Molecular Sciences, University of Oslo, 0315, Oslo, Norway.

[3] Zernike Institute of Advanced Materials, University of Groningen, Nijenborgh 3, 9747 AG Groningen, the Netherlands.

[4] MESA+ Institute for Nanotechnology, University of Twente, 7500 AE Enschede, the Netherlands.

[5] Max Planck Institute for Chemical Energy Conversion, Stiftstraße 34-36, 45470, Mülheim an der Ruhr, Germany.

[6] Department of Biochemistry and Molecular Biology, The Pennsylvania State University, University Park, Pennsylvania 16802, United States

[7]NMR Spectroscopy, Bijvoet center for Biomolecular Research, Utrecht University, Padualaan 8, 3584 CH, Utrecht, The Netherlands.

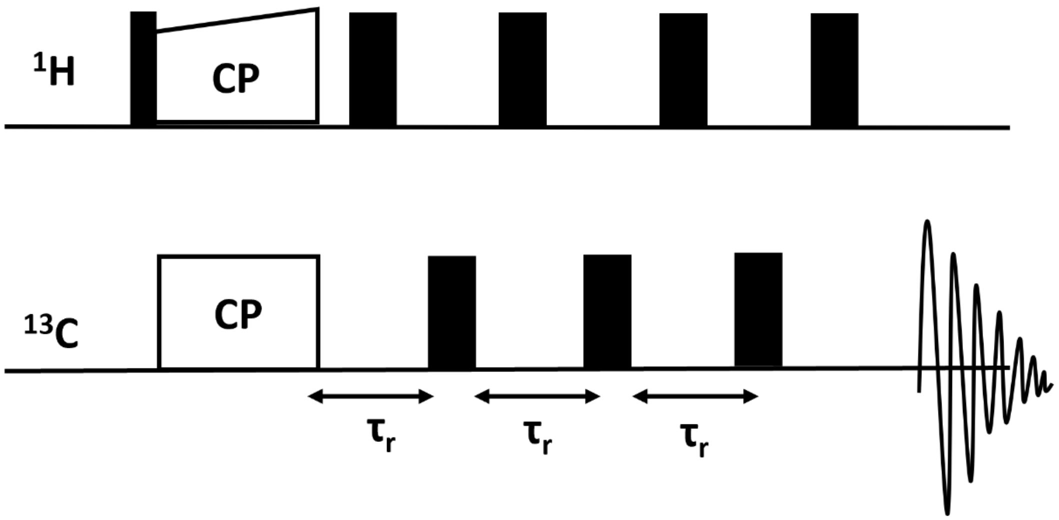

Figure S1. pulse sequence used for ^{13}C {^{1}H} REDOR NMR.^{13}C is used as the observe channel and refocusing π pulses are applied on the ^{1}H channel. The ^{1}H π/2 pulse is 1.5 μs and the CP contact time is set to 2 ms. ^{13}C π pulse is set to 10 μs. The pulse sequence used has been implemented according to Cui *et al.*[1]

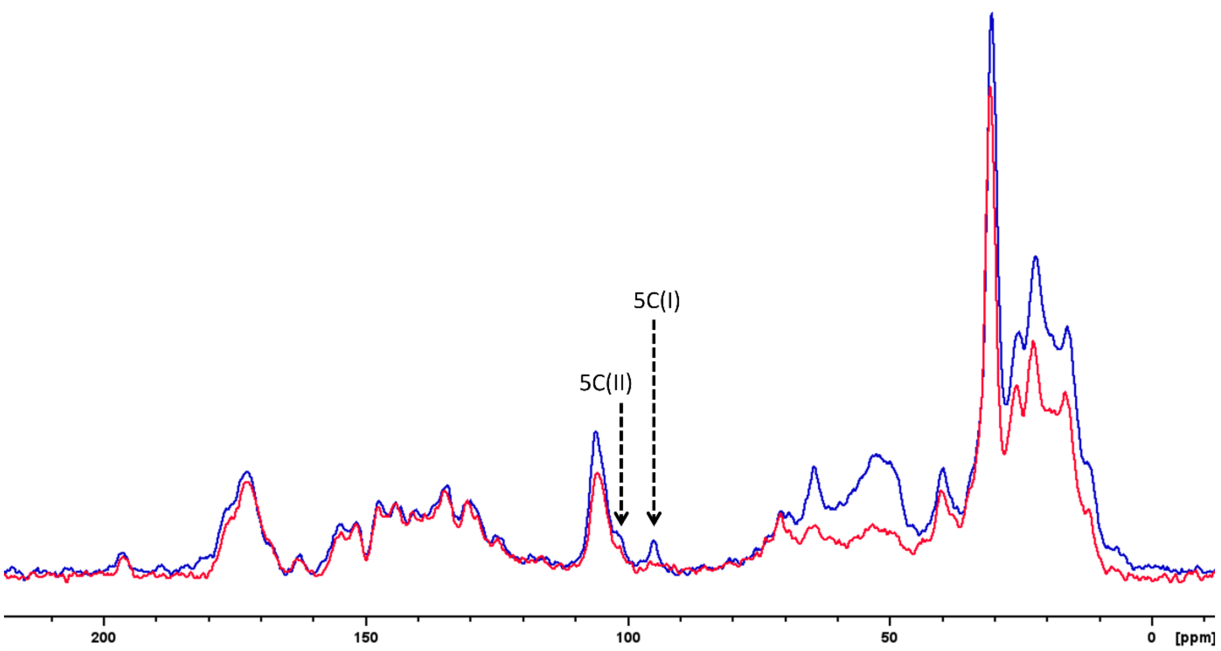

Figure S2. $^{13}C\{^{1}H\}$ REDOR S_0 (blue) and S_R (red) spectra of *bchQ* chlorosomes measured at 288 K spinning at 50 kHz in a 1.3 mm rotor. The two dashed arrows indicate the two conformations that are observed in 1-D spectra for the 5C position. The analysis of the REDOR curve mentioned in the main text is performed for the 5C(I) signal which represents ~ 70% of the total signal intensity for both components. The 5C(II) signal overlaps with the peak corresponding to 10, 15, and 20 carbon positions and REDOR works best for isolated spin systems. Therefore 5C(I) was chosen for the analysis. From the S_0 and S_R spectra, the dephasing ratios S_R/S_0 for the carbonyl (CO), methyl (CH_3) and methine (CH) groups in the above spectra are also comparable to the dephasing ratios of 90 %, 47 % and 8 % for the CO, CH_3 and CH groups observed for L valine by Ishii *et al.*[2]

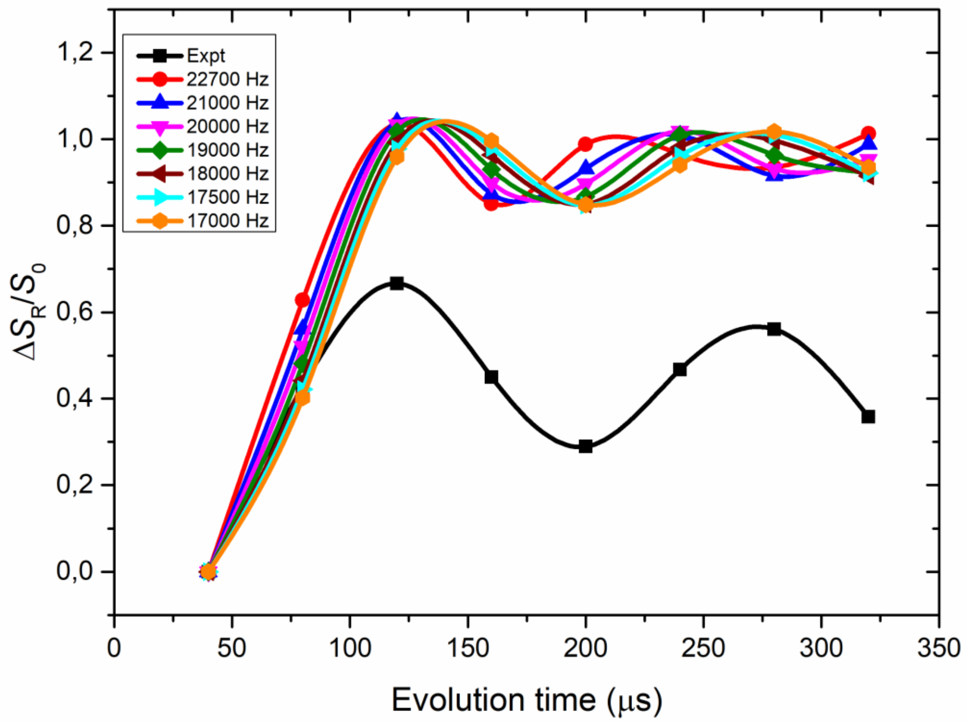

Figure S3. $^{13}C\{^{1}H\}$ dephasing curve as a function of time. The black cubic spline represents the experimentally obtained data set. The colored dephasing curves were obtained by simulation using SIMPSON for different distances. The best match of the dipolar coupling strength to the experimental dephasing frequency is found for a dipolar coupling strength of 17.5 kHz, based on the first minimum and the second maximum of the dephasing curve (cyan spline).

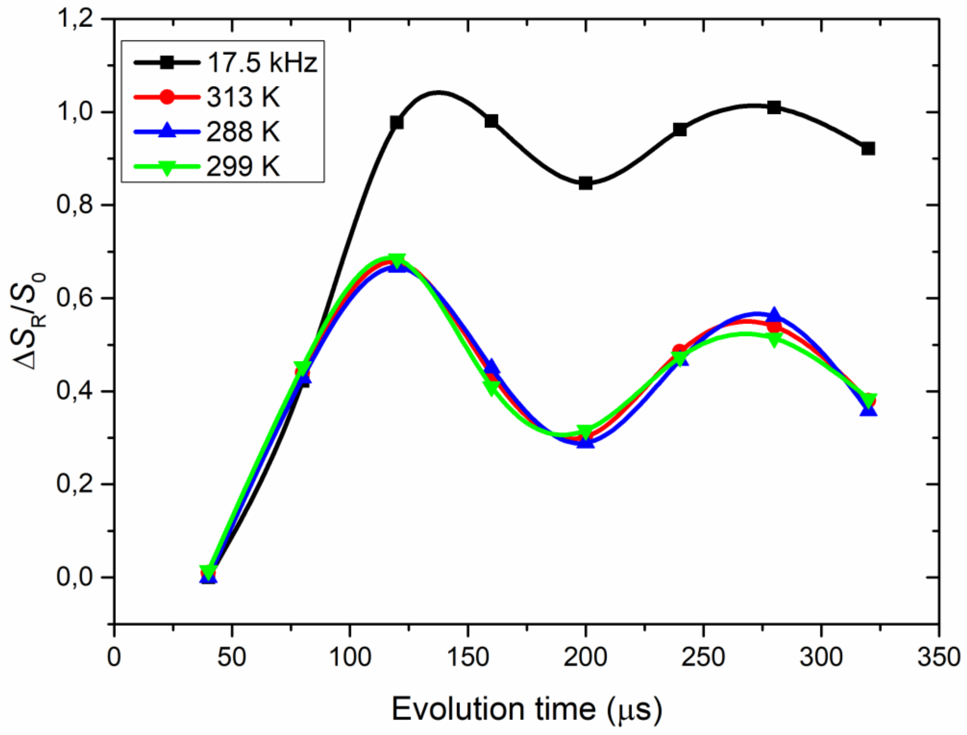

Figure S4. REDOR $^{13}C\{^{1}H\}$ dephasing curve as a function of time at 288 K, 299 K, and 313 K and its close match with the dipolar coupling strength obtained from SIMPSON[3] simulation for 17.5 kHz.

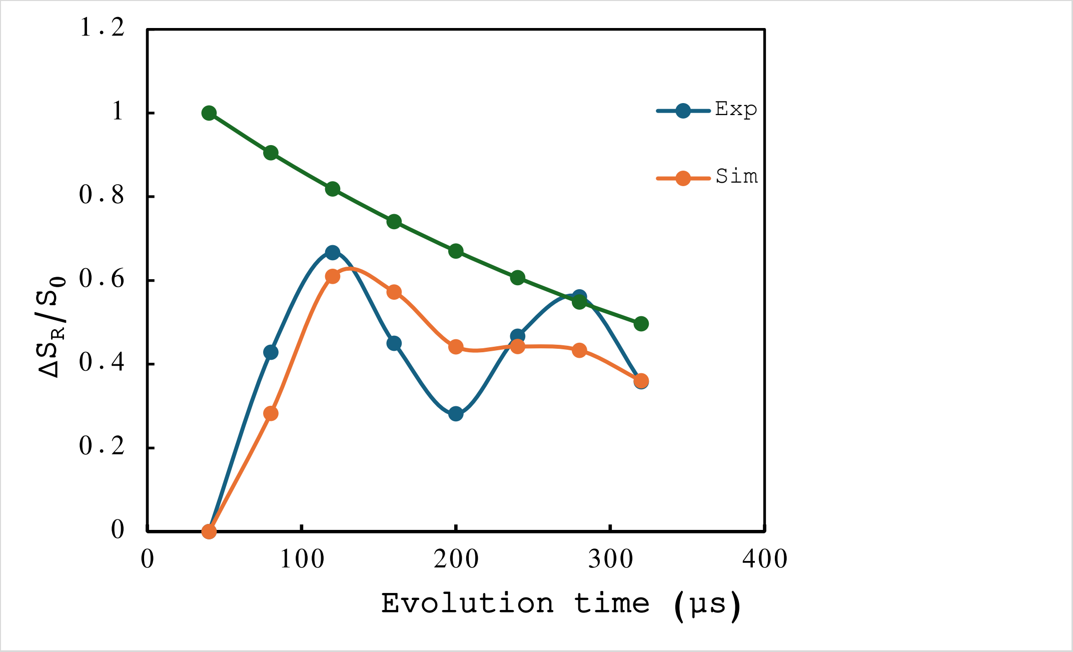

Figure S5: Best fit of the model data $m(t_i)$ for a coupling strength of 17500 Hz to the experimental REDOR $^{13}C\{^{1}H\}$ dephasing data set $d(t_i)$. As before, we employ a cubic spline interpolation for visualisation purposes. In addition to a time-independent scaling factor c, we have introduced a time-dependent exponentially decaying filter exp(-t/a) and minimized $res(t_i)=d(t_i)-c*exp(-t/a)*m(t_i)$ for both c and a, and in the least squares sense. The best fit was determined for a=400 **μ**s and c=0.775, with a RMSD of 0.0970. Applying the same procedure for model data for coupling strengths of 17 kHz and 18 kHz provides c=0.776 and RMSD=0.1014 and c=0.772 and RMSD=0.0939, respectively. We note that the same exponential filter was determined optimal for these values.

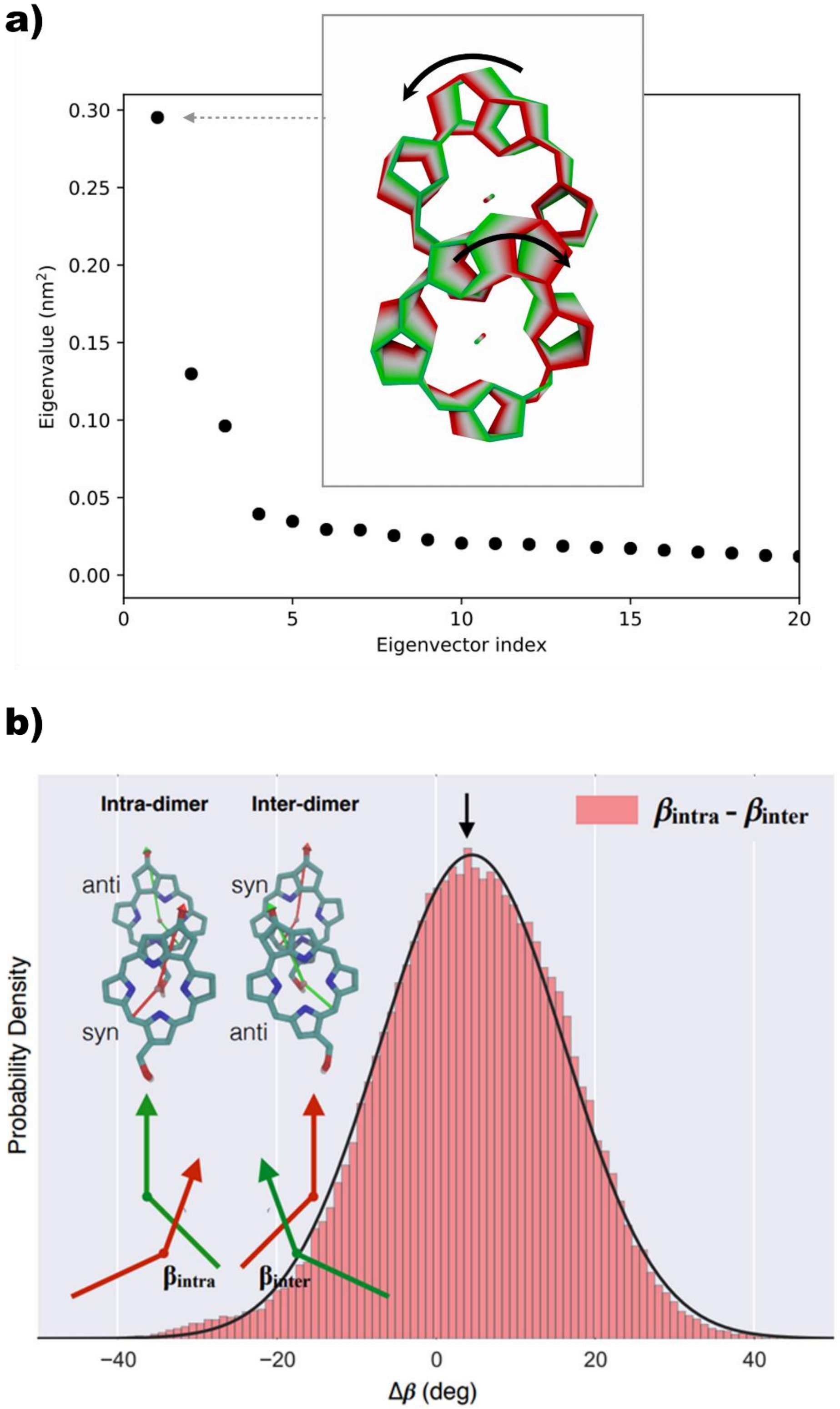
a)
0.30
0.25
0.20
0.15
0.10
0.05
0.00
Eigenvalue (nm²)
0
5
10
15
20
Eigenvector index
b)
Intra-dimer
Inter-dimer
anti
syn
syn
anti
βintra
βinter
βintra - βinter
Probability Density
−40
−20
0
20
40
Δβ (deg)

Figure S6. a) Principal component analysis (PCA) results for a dimer trajectory that was randomly selected from a tube at T=300 K. The notion of chlorosomes as a plastic molecular crystal or rotator phase is based on the observation of persistent, dominant rotational (or librational) motion exhibited by the BChl macrocycle. The graph shows the magnitude of the eigenvalue plotted against the eigenvector index. Additionally, the inset displays the first mode, with arrows indicating the rotational directions and green representing earlier and red later stages, respectively. Only a few modes are identified as significant based on the magnitude of the eigenvalues. The primary rotational motion perturbs the hydrogen bonding network, as the rotation can either break or form hydrogen bonds between moieties in neighboring macrocycles.[5] The other modes correspond to experimentally observed side chain fluctuations and tail dynamics described in the main text. Figure reproduced from Li *et al*.[4] Copyright (2022) Elsevier.

Figure S6. b) The *syn-anti* dimer is the structural unit of the stack, with the orientations between *syn-anti* and *anti-syn* pairs alternating along the stacking direction. The distribution of the relative angle, i.e. the difference $\beta_{intra} - \beta_{inter}$ between neighboring intradimer and interdimer rotation angles, see the inset for a definition of β_{intra} and β_{inter}, is due to dynamic heterogeneity and was extracted from an MD trajectory for a (30, 30) BChl *c* system simulated at a temperature of 300 K.

The important part of the sampling distribution obtained from relatively short molecular simulation between 500 ps and 1 ns lies between -20° to 20° around the average value, spanning about 40° in total, a value that is in very good agreement with the effective $\theta=48^\circ \pm 4^\circ$ angle obtained from the two site model. Figure 8 of Li *et al*,[5] showing the relative angle distribution at a much lower temperature of 50 K, illustrates the persistence of the

dynamics towards low temperatures. While the distribution width is conserved at T=50 K compared to T=300 K, the bimodal nature of the angle distribution signals an undersampling in the considered 500 ps to 1 ns interval due to a weaker thermal driving force, which will not be observed on the timescale of NMR. Figure reproduced from Li *et al.*[5] Copyright (2018) American Chemical Society.

REDOR script used for simulations

```
spinsys {
  channels 1H 13C
  nuclei   1H 13C
  dipole 1 2 -17500 0 98.58 -25.11

}

par {
variable index          1
np                      8
spin_rate               50000
proton_frequency        750e6
start_operator          I2x
detect_operator         I2p
method                   direct
crystal_file             rep320
gamma_angles             32
sw                      spin_rate/2
variable tsw            1e6/sw
```

```
verbose                 1101
variable tr             1e6/spin_rate
variable tr2             0.5e6/spin_rate
variable rf             166000
variable t180           0.5e6/rf
}

proc pulseq {} {
global par

set t180 [expr 0.5e6/$par(rf)]
set tr2 [expr 0.5e6/$par(spin_rate)-$t180]
reset
delay $tr2
pulse $t180 0 x $par(rf) x
delay $tr2
pulse $t180 0 x $par(rf) y
store 1
reset
acq
delay $tr2
pulse $t180 0 x $par(rf) y
delay $tr2
pulse $t180 $par(rf) x 0 x
prop 1
store 2
acq
```

```
for {set i 2} {$i < $par(np)} {incr i} {
reset
prop 1
prop 2
prop 1
  store 2
  acq
}
}


proc main {} {
  global par

  set f [fsimpson]
  fsave $f $par(name),$par(index).fid
}
```

REFERENCES

(1) Cui, J.; Olmsted, D. L.; Mehta, A. K.; Asta, M.; Hayes, S. E. NMR Crystallography: Evaluation of Hydrogen Positions in Hydromagnesite by 13C1H REDOR Solid-State NMR and Density Functional Theory Calculation of Chemical Shielding Tensors. *Angewandte Chemie International Edition* **2019**, *58* (13), 4210–4216. https://doi.org/10.1002/anie.201813306.

(2) Ishii, Y.; Wickramasinghe, N. P.; Chimon, S. A New Approach in 1D and 2D 13C High-Resolution Solid-State NMR Spectroscopy of Paramagnetic Organometallic Complexes by Very

Fast Magic-Angle Spinning. *J. Am. Chem. Soc.* **2003**, *125* (12), 3438–3439. https://doi.org/10.1021/ja0291742.

(3) Bak, M.; Rasmussen, J. T.; Nielsen, N. C. SIMPSON: A General Simulation Program for Solid-State NMR Spectroscopy. *Journal of Magnetic Resonance* **2000**, *147* (2), 296–330. https://doi.org/10.1006/jmre.2000.2179.

(4) Li, X.; Buda, F.; De Groot, H. J. M.; Sevink, G. J. A. The Role of Chirality and Plastic Crystallinity in the Optical and Mechanical Properties of Chlorosomes. *iScience* **2022**, *25* (1), 103618. https://doi.org/10.1016/j.isci.2021.103618.

(5) Li, X.; Buda, F.; de Groot, H. J. M.; Sevink, G. J. A. Contrasting Modes of Self-Assembly and Hydrogen-Bonding Heterogeneity in Chlorosomes of Chlorobaculum Tepidum. *J. Phys. Chem. C* **2018**, *122* (26), 14877–14888. https://doi.org/10.1021/acs.jpcc.8b01790.
